# The In Vitro Anti-Cancer Activities and Mechanisms of Action of 9-Methoxycanthin-6-one from *Eurycoma longifolia* in Selected Cancer Cell Lines

**DOI:** 10.3390/molecules27030585

**Published:** 2022-01-18

**Authors:** Nurhanan Murni Yunos, Nor Datiakma Mat Amin, Muhammad Haffiz Jauri, Sui Kiong Ling, Nor Hasnida Hassan, Nor Jannah Sallehudin

**Affiliations:** 1 Bioactivity Programme, Natural Products Division, Forest Research Institute Malaysia, Kepong 52109, Malaysia; nordatiakma@frim.gov.my (N.D.M.A.); norjannah@frim.gov.my (N.J.S.); 2 Phytochemistry Programme, Natural Products Division, Forest Research Institute Malaysia, Kepong 52109, Malaysia; haffiz@frim.gov.my (M.H.J.); lingsk@frim.gov.my (S.K.L.); 3 Biotechnology Programme, Forestry Biotechnology Division, Forest Research Institute Malaysia, Kepong 52109, Malaysia; hasnida@frim.gov.my

**Keywords:** *Eurycoma longifolia*, 9-methoxycanthin-6-one, tissue culture, cancer cell lines, apoptosis, proteomics

## Abstract

An alkaloid compound from the hairy root culture of *Eurycoma longifolia* has been isolated and characterised as 9-methoxycanthin-6-one. The aims of these studies were to investigate the in vitro anti-cancer activities of 9-methoxycanthin-6-one against ovarian cancer (A2780, SKOV-3), breast cancer (MCF-7), colorectal cancer (HT29), skin cancer (A375) and cervical cancer (HeLa) cell lines by using a Sulphorhodamine B assay, and to evaluate the mechanisms of action of 9-methoxycanthin-6-one via the Hoechst 33342 assay and proteomics approach. The results had shown that 9-methoxycanthin-6-one gave IC_50_ values of 4.04 ± 0.36 µM, 5.80 ± 0.40 µM, 15.09 ± 0.99 µM, 3.79 ± 0.069 µM, 5.71 ± 0.20 µM and 4.30 ± 0.27 µM when tested in A2780, SKOV-3, MCF-7, HT-29, A375 and HeLa cell lines, respectively. It was found that 9-methoxycanthin-6-one induced apoptosis in a concentration dependent manner when analysed via the Hoechst 33342 assay. 9-methoxycanthine-6-one were found to affect the expressions of apoptotic-related proteins, that were proteins pyruvate kinase (PKM), annexin A2 (ANXA2), galectin 3 (LGAL3), heterogeneous nuclear ribonucleoprotein A1 (HNRNP1A1), peroxiredoxin 3 (PRDX3), and glyceraldehyde-3-phosphate dehydrogenase (GAPDH) from the differential analysis of 2-DE profiles between treated and non-treated 9-methoxycanthine-6-one. Proteins such as acetyl-CoA acyltransferase 2 (ACAA2), aldehyde dehydrogenase 1 (ALDH1A1), capping protein (CAPG), eukaryotic translation elongation factor 1 (EEF1A1), malate dehydrogenase 2 (MDH2), purine nucleoside phosphorylase (PNP), and triosephosphate isomerase 1 (TPI1) were also identified to be associated with A2780 cell death induced by 9-methoxycanthine-6-one. These findings may provide a new insight on the mechanisms of action of 9-methoxycanthin-6-one in exerting its anti-cancer effects in vitro.

## 1. Introduction

Cancer cases are still on the rise in both developed and developing countries, and it is predicted that 14 million new cancer cases will emerge by 2035, representing almost 60% of the global cancer incidence, as compared to 6.7 million new cancer cases (47.5% of all cancers) reported in 2012 [1]. Cancer may affect more than a hundred types of cells from more than sixty different organs in the body [2]. Hence, there is still a dire need to search for new and alternative treatments for treating various type of cancers. Chemotherapy is still the main modality of treatments for advanced stage of cancers. However, chemotherapy cannot cure various types of cancers via single treatment, and also has drug resistance and toxicity side effects problems. Cancer arises due to malfunctions of certain genes and their translated proteins that are related to the over cell proliferations, divisions and growth and/or suppressions of apoptosis-related proteins [3]. Apoptosis or programmed cell death is a process involving expressions of certain genes and proteins which signal the cells to commit suicide in order to eliminate the potential cancer cells’ development in our body [4]. Most of the cancer cells malfunction in apoptosis and many anti-cancer therapeutic agents were found to re-induce the apoptosis mechanism and produce a better outcome to prevent cancer cells’ proliferation [5]. Thus, phytochemical(s) that enable the re-inducement of the apoptosis mechanism in the cancer cells may have the potential to be studied further towards development of anti-cancer therapeutic agents.

*Eurycoma longifolia* is a plant species that belongs to the family of Simaroubaceae. It is one of the most well-known herbal folk medicines in southeast Asia [2]. *E. longifolia* is identified locally as ‘Tongkat Ali’ in Malaysia, ‘Pasak bumi’ or ‘Bidara Pahit’ in Indonesia and ‘Piak’, ‘Tung saw’, ‘Ian-don’ in Thailand and ‘Tho nan’ (Laotian) [2,6]. The root of *E. longifolia*, which is known for its bitter taste, is most sought after because of its various traditional uses for health maintenance, and is also scientifically investigated to have many medicinal properties. Thus, *E. longifolia* was named as ‘Cay ba binh’, translated as a tree of a hundred maladies, in Vietnam [7]. For examples, the decoction prepared from the roots were claimed to be used as a tonic after childbirth, to treat intermittent fever and to relieve pain in the bones [6,8]. The poultice was prepared for treating headaches, wounds, ulcers and syphilitic sores [6]. The phytochemicals from the roots of *E. longifolia* had been reported to have anti-ulcer, anti-bacterial, cytotoxic/anti-cancer, anti-parasitic, anti-malarial, anti-pyretic and aphrodisiac properties [2,9]. Different groups of compounds, such as quassinoids, alkaloids, bipheylneolignans, triterpenes and steroids, are among the phytochemicals that have been reported to be present in different plant parts of *E. longifolia* [10,11]. 

Most of the compounds from *E. longifolia* reported to have anti-cancer effects on the tested cancer cell lines were from quassinoids, alkaloids and triterpenes. Quassinoids are among the major compounds detected in most species belonging to Simaroubaceae, including *E. longifolia* [12]. Among the quassinoids reported to have in vitro anti-cancer effects are eurycomanone, eurycomalactone, 14,15 β-dihydroxyklaineanone, 6-dehydroxylongilactone and others that will be mentioned later. Eurycomanone was reported to have anti-cancer effects in breast [10,13], colon, fibrosarcoma, lung and melanoma cancer cell lines [13]. Eurycomalactone was reported to have anti-cancer effects in murine lymphocytic leukemia (P388) and epidermoid (KB) [14], lung cancer (A-549), breast cancer (MCF-7) [10] and colon (26-L5), melanoma (B16-BL6), lung (LLC and A-549) cancer cell lines [15]. 14,15 β-dihydroxyklaineanone and 6-dehydroxylongilactone were reported to have anti-cancer effects in murine lymphocytic leukemia (P388), epidermoid (KB) [14], lung cancer (A-549) and breast cancer (MCF-7) cell lines [10]. Other quassinoids had also been reported to have anti-cancer effects in respective cancer cell lines. Longilactone was active in murine lymphocytic leukemia (P388), epidermoid (KB) cancer cell lines and lung cancer (A-549) [10]. Pasakbumin C demonstrated strong anti-cancer activities in lung cancer (A-549) cell line. Pasakbumin B and pasakbumin C had also exhibited strong anti-activities in human breast cancer (MCF-7) cell line [10]. Another two quassinoids (11-dehydroklaineanone, 5,6-dehydroeurycomalactone) and seven tirucallane-type triterpenes (niloticin, dihydroniloticin, piscinidol A, bourjotinolone A, 3-episalin A, melianone, hispidone) from the woods of *E. longifolia* were found to be active when treated in murine lymphocytic leukemia (P388) and epidermoid (KB) cancer cell lines [14]. 

Alkaloids are also among the compounds that had been mostly isolated from *E. longifolia* [11]. Among alkaloids from *E. longifolia* that had been reported to have anti-cancer effects were 9-methoxycanthin-6-one, canthin-6-one [13,16], 9-methoxycanthin-6-one-*N*-oxide, 9-hydroxycanthin-6-one, 9-hydroxycanthin-6-one-*N*-oxide [13]. 9-methoxycanthin-6-one had been reported to have anti-cancer effects in breast [13,16,17], lung [13,16], colon, fibrosarcoma, melanoma, epidermoid [13], prostate (DU-145) and ovarian (CaOV-3) cancer cell lines [17]. Canthin-6-one had been reported to have anti-cancer effects in lung (A-549) and breast (MCF-7) cancer cell lines [16]. 9-methoxycanthin-6-one-*N*-oxide, 9-hydroxycanthin-6-one, 9-hydroxycanthin-6-one-*N*-oxide had been reported to have anti-cancer effects in breast, colon, fibrosarcoma, lung, melanoma and epidermoid cancer cell lines [13].

Natural products, including phytochemicals, still remain among the main sources in discovering the anti-proliferative agents for cancer treatments due to their diversity in chemical structures [18]. Over the time frame from around 1981 to 2019, 64.9% either natural products, or those which mimicked or were based on natural products in one form or another, had been developed into cancer drugs [18]. In these studies, our team had pursued on the mechanisms of action studies on 9-methoxycanthin-6-one because this information is still lacking, whereas many mechanisms of action studies had been reported on quassinoids such as eurycomanone [19]. Apoptotic and proteomics analyses on a selected cancer cell line treated with 9-methoxycanthin-6-one may provide us with some evidences on the mechanisms of action of this compound in executing cancer cell death. These studies were among the first steps in determining the anti-cancer potential of 9-methoxycanthin-6-one before embarking on drug discovery and pre-clinical studies.

## 2. Results

### 2.1. Identification of 9-Methoxycanthin-6-one

**Figure 1:**
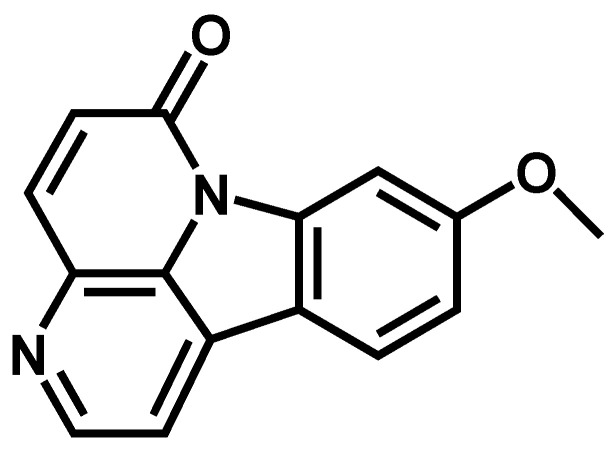
Structure of 9-methoxycanthine-6-one.

*9-methoxycanthin-6-one* (**1**), a yellow amorphous powder, and its molecular formula was determined as C_15_H_10_N_2_O_2_ by EIMS at *m/z* 251.3 [M + H]^+^, ^1^H and ^13^C-NMR spectra (as shown in Table 1). The ^1^H-NMR spectrum of (**1**), showed a pair of ortho-coupled signals at 6.99 (1H, d, *J* = 2.4 Hz) and δ 7.98 (1H, d, *J* = 2.4 Hz), δ 8.23 (1H, d, *J* = 5.2 Hz) and 8.78 (1H, d, *J* = 5.2 Hz), corresponding to four aromatic protons of monosubtituted indole moiety. A signal resolved at δ 4.04 (3H, s) indicated methoxy protons in compound (**1**). The ^13^C-NMR peak assignments showed a methoxy carbon resonated at δ 56.2, a signal at δ 163.41 attributed to carbonyl carbon (C-6), and seven signals, δ 115.66 (C-1), δ 143.81 (C-2), δ 138.05 (C-4), δ 129.69 (C-5), δ 101.45 (C-8), δ 114.76 (C-10) and δ 123.95 (C-12), corresponding to seven aromatic carbons. The known constituent (**1**) was identified by comparison of its spectral data of ^1^H, ^13^C-NMR and MS with those reported in the literature [20,21]. The structure of 9-methoxycanthin-6-one (**1**) is shown in Figure 1.

### 2.2. In Vitro Anti-Cancer Activities of 9-Methoxycanthine-6-one on Selected Cancer Cell Lines 

**Figure 2:**
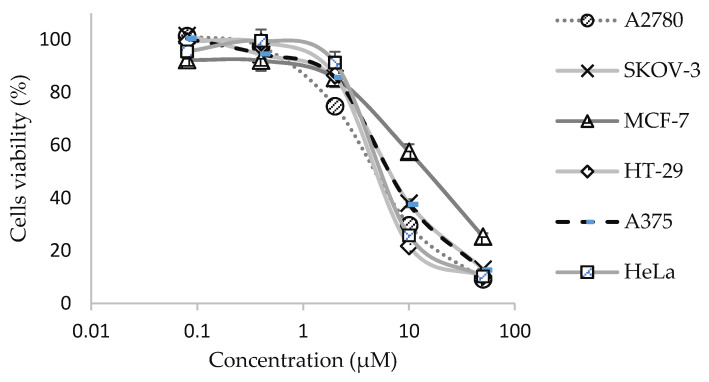
Dose–response curves of 9-methoxycanthin-6-one when tested in a panel of cancer cell lines (ovarian cancer (A2780, SKOV-3), breast cancer (MCF-7), colorectal cancer (HT-29), cervical cancer (HeLa) and skin cancer (A375)). Data represent the average percentage of cell viability ± SEM, *n* = 9. The curves for SKOV-3 and A375 are overlapping.

The in vitro anti-cancer effects of 9-methoxycanthin-6-one were evaluated against six different cancer cell lines: A2780, SKOV-3, MCF-7, HT29, A375 and HeLa. Cisplatin and paclitaxel were also evaluated on their in vitro anti-cancer effects for comparison studies. 9-methoxycanthin-6-one gave significant in vitro anti-cancer effects, with IC_50_ values ranging from 3.79 ± 0.069 μM to 15.09 ± 0.99 μM, as illustrated in the dose–response curves in Figure 2 and tabulated in Table 2. Whereas, cisplatin and paclitaxel exerted better in vitro anti-cancer activities with the range of IC_50_ between 1.38 ± 0.037 to 3.58 ± 0.14 μM and between 0.018 ± 0.00011 to 0.38 ± 0.012 μM, respectively (Figure 2, Table 2). However, 9-methoxycanthin-6-one showed a lower cytotoxicity effect as compared with cisplatin when tested in the cardiomyocyte (H9C2) cell line (Table 2).

### 2.3. Effect of 9-Methoxycanthin-6-one on Apoptosis 

**Figure 3:**
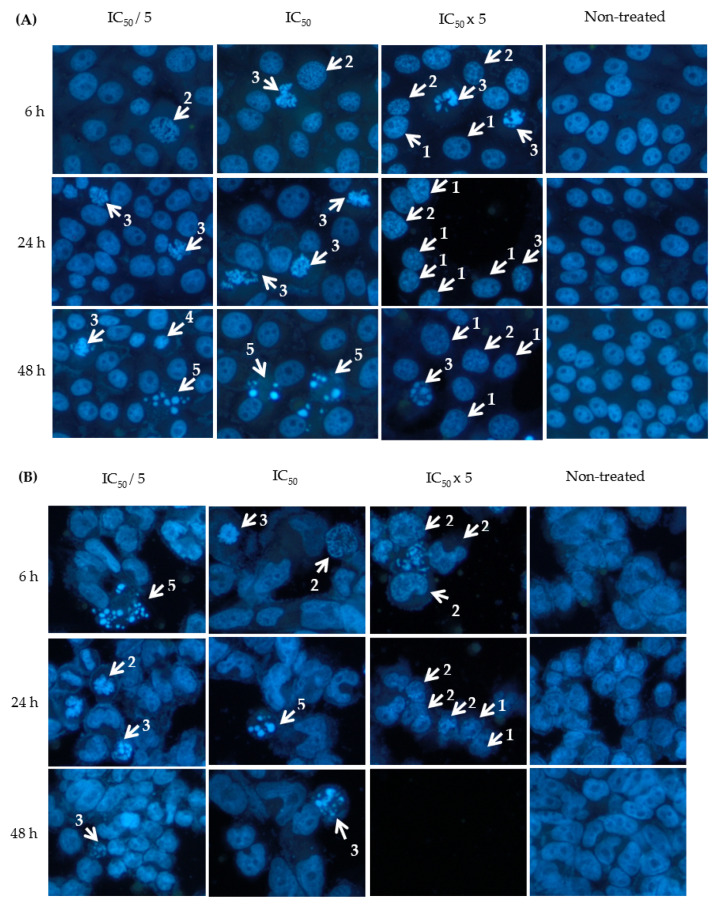

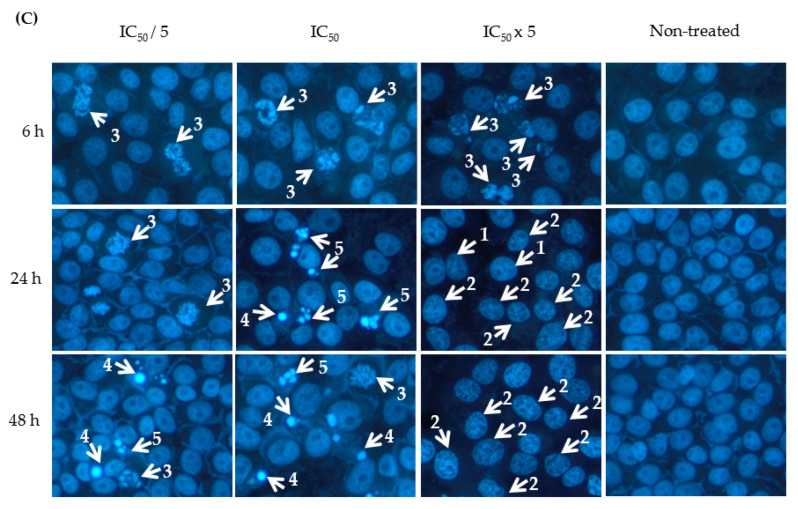
Visualisation of apoptotic cells under the fluorescence microscope when cancer cells, (**A**) HT-29, (**B**) A2780 and (**C**) SKOV-3 were treated with 9-methoxycanthin-6-one at different concentrations (IC_50_/5, IC_50_ and IC_50_ × 5) and incubation times (6, 24 and 48 h). Cells that underwent apoptosis showed morphology changes such as (1) minimal chromatin condensation, (2) extensive chromatin condensation, (3) compaction of chromatin to a dense mass at the periphery of nuclei, (4) compaction of chromatin into a dense ball, and (5) apoptotic bodies. Magnification: ×200.

The Hoechst 33342 assay was employed to study the effect of 9-methoxycanthin-6-one on apoptosis at different concentrations and incubation times in HT-29, A2780 and SKOV-3 cancer cell lines. Cells that undergo apoptosis have morphology changes, such as chromatin condensation, DNA fragmentation, cell shrinkage, cell membrane blebbing and formation of apoptotic bodies [4]. As shown in Figure 3, the formation of chromatin condensation and apoptotic bodies was observed when HT-29, A2780 and SKOV-3 cancer cells were treated with 9-methoxycanthin-6-one. The percentage of apoptotic indices were shown in Table 3. 9-methoxycanthin-6-one significantly increased the percentage of apoptotic indices in HT-29, A2780 and SKOV-3 cells when the concentrations of 9-methoxycanthin-6-one increased. However, 9-methoxycanthin-6-one treated at different incubation times (6 h, 24 h and 24 h) had no significant difference with the percentage of apoptotic indices when the cancer cells were treated at the same concentration. 

### 2.4. 2-DE Analysis of 9-Methoxycanthin-6-one-treated and Non-Treated A2780 Ovarian Cancer Cells 

**Figure 4:**
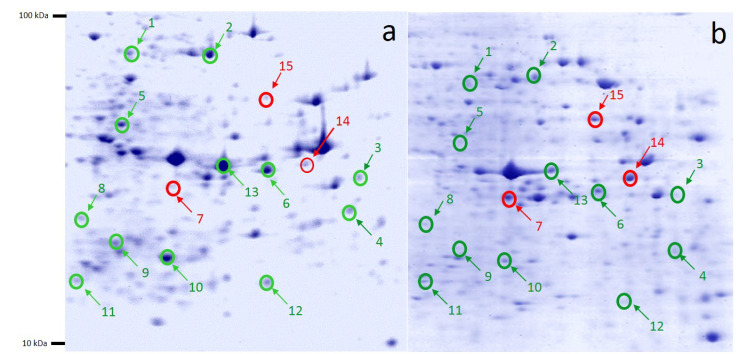
Representative of 2-DE gel profiles of (**a**) non-treated and (**b**) 9-methoxycanthin-6-one-treated A2780 ovarian cancer cells. Fifteen protein spots that were differentially expressed (anova *p* < 0.05) and showed an average expression ratio of at least 1.5 fold in both gels. Green labels represent proteins that were suppressed and red labels represent proteins that were expressed when A2780 cancer cells were treated with 9-methoxycanthin-6-one.

Approximately more than 922 spots were detected in each Coomassie brilliant blue-stained gels. Fifteen differentially expressed protein spots, derived from the 9-methoxycanthine-6-one-treated and non-treated A2780 cell line, with the selection criteria of a spot intensity of more than 1.5 fold changes and anova *p* < 0.05 2DE gels, were selected, picked and subjected for protein identifications using MALDI-TOF-MS/MS. The protein spots that were selected for protein identifications on the 2-DE profiles were labelled in Figure 4. From Figure 4, 12 proteins were suppressed and 3 proteins were expressed after A2780 ovarian cancer cell line was treated with 9-methoxycanthin-6-one. 

### 2.5. Protein Identifications 

MALDI-TOF/TOF tandem mass spectrometry analysis and a Mascot database search were performed for the 15 proteins that were differentially expressed when the A2780 ovarian cancer cells were treated with 9-methoxycanthine-6-one (as shown from Figure 4). The protein name, Mascot accession number, estimated and theoretical pI/Mwt, sequence coverage and mascot score were listed in Table 4. The proteomics dataset was introduced to Ingenuity Pathway Analysis (IPA) for core biochemical pathway analysis. In IPA, the protein was classified according to their biological functions, mechanisms and relevant protein–protein interactions in the form of a global protein interaction network. 

### 2.6. Proteins’ Interaction Analysis 

**Figure 5:**
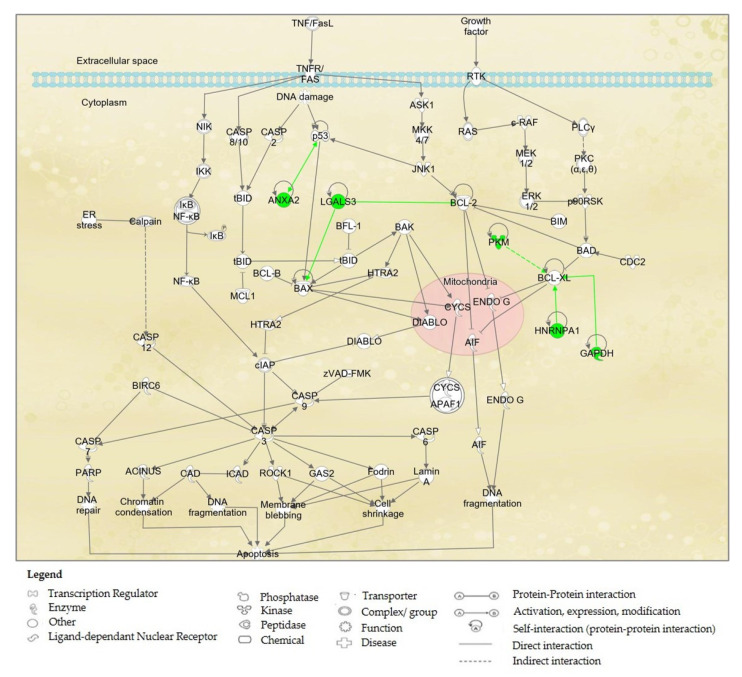
Diagram shows protein–protein interactions in apoptosis signaling pathway and the involvement of five of the differentially expressed proteins from A2780 ovarian cancer cells treated with 9-methoxycanthine-6-one. Proteins that were labelled in green were proteins that were suppressed by 9-methoxycanthine-6-one.

The 15 identified proteins listed from Table 4 were further analysed for protein–protein interactions in IPA. IPA via its Ingenuity^®^ Knowledge Base platform had classified these proteins according to their involvements in certain biochemical/ canonical pathways. Based on IPA analysis, 5 proteins, namely PKM, ANXA2, LGAL3, HNRNP1A1, PRDX3 and GAPDH, were found to be associated with the apoptosis signaling pathway in A2780 ovarian cancer cell line. The involvement of these proteins in the apoptosis signaling pathway may explain the mechanisms of the action of 9-methoxycanthine-6-one when treated in the A2780 cancer cell line, and is shown in Figure 5. 

**Figure 6:**
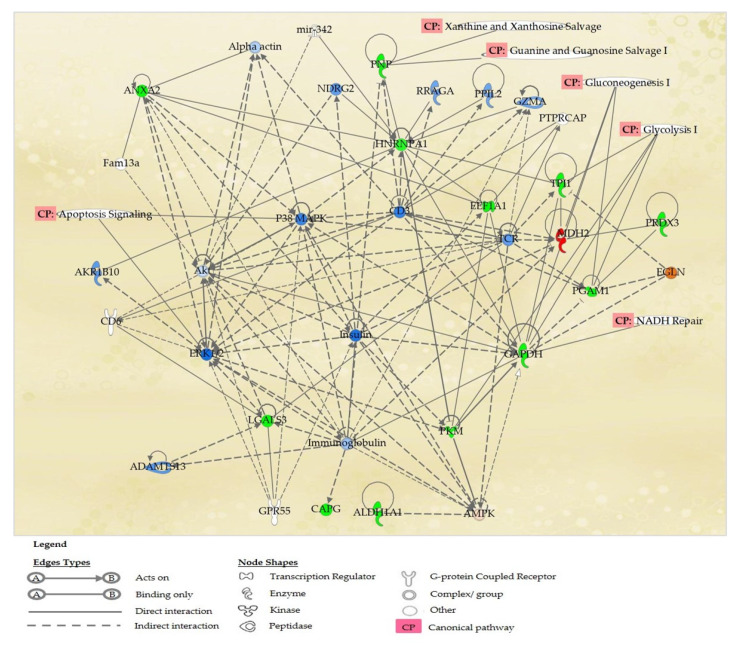
IPA graphical representation of the molecular relationships between differentially expressed proteins in A2780 cell lines treated with compound 9-methoxycanthine-6-one. The network is displayed graphically as nodes (proteins) and edges (the biological relationships between the nodes). Nodes in red indicate expressed proteins, nodes in green represent suppressed proteins, nodes in blue indicate predicted inhibition of expression, nodes in orange indicate predicted activation of proteins, and nodes without colours indicate unaltered expression in the network. Various shapes of the nodes represent the functional class of the proteins. The different arrow shapes represent different types of interactions. Edges are displayed with various labels that describe the nature of the relationship between the nodes.

Thirteen proteins, namely ALDH1A1, ANXA2, CAPG, EEF1A1, GAPDH, HNRNPA1, LGALS3, MDH2, PGAM1, PKM, PNP, PRDX3 and TPI1, were primarily associated in the top networks of “Carbohydrate Metabolism, Cellular Movement and Cellular Response to Therapeutics”, which lead towards the execution of ovarian cancer cell death. These networks involved these canonical pathways: glycolysis I, gluconeogenesis I, NADH Repair, Xanthine and Xanthosine Salvage, and Guanine and Guanosine Salvage I (Figure 6). Proteins NVL and ACAA2 that were not involved in the top network and apoptosis signaling pathway, were found to be involved in the network of “Cell-To-Cell Signaling and Interaction, Nervous System Development and Function, and RNA Post-Transcriptional Modification”, that may also lead to ovarian cancer cell death. 

Figure 6 showed the top canonical pathways affected after the A2780 ovarian cancer cell line was treated with 9-methoxycanthine-6-one. The proteins involved in the respective pathways were listed in Table 5. IPA results showed that Glycolysis I is the most effected canonical pathway when 9-methoxycanthine-6-one treated the A2780 ovarian cancer cell line.

## 3. Discussion

From previous studies, 9-methoxycanthin-6-one was detected to be present in wild-derived roots [13,22] as well as in the tissue culture [23,24,25] of *E. longifolia.* It is interesting to note that plant tissue cultures can be used as an alternative source, rather than wild and cultivated plant sources, to isolate the phytochemical(s) of interests in a short period of time with an improved yield [26]. In our studies, the yield of 9-methoxycanthin-6-one that was isolated from hairy roots’ culture was 0.43% (*w/w*). Other studies have also supported this finding in which the yield of 9-methoxycanthin-6-one (1) was higher in *E. longifolia* tissue cultures than in *E. longifolia* roots collected from the wild. The yield of 9-methoxycanthin-6-one obtained from the callus and tissue culture ranged from 0.41% to 0.71% (*w/w*) [23,24,25], as compared to the yield of 9-methoxycanthin-6-one from the wild, that was ranged from 0.009% to 0.045% (*w/w*) [13,22]. It may be noted that other anti-cancer compounds, such as camptothechin and paclitaxel, had also been successfully isolated from the *Camptotheca acuminata* hairy roots and *Taxus cuspidate* callus, respectively [27,28].

The in vitro anti-cancer analysis had also shown that this compound had anti-cancer potential, since the IC_50_ values were less than 50 µM [29]. Previous findings had shown that 9-methoxycanthin-6-one (1) possessed in vitro anti-cancer effects in breast, colon, fibrosarcoma, lung and melanoma cancer cell lines and, [13] with another study, had also supported the anti-cancer activities of 9-methoxycanthin-6-one in lung cancer (A-549) and breast cancer (MCF-7) cell lines [16]. 9-methoxycanthin-6-one was shown to be active in breast (MCF-7), prostate (DU-145) and ovarian (CaOV-3) cancer cell lines [17]. To the best of our knowledge, other than ovarian, breast and colorectal cancer cell lines, 9-methoxycanthin-6-one was the first to be reported to have in vitro anti-cancer effects in cervical cancer (HeLa) and skin cancer (A375) cell lines. Thus, all of these findings may indicate that 9-methoxycanthin-6-one has a wider spectrum of in vitro anti-cancer activities, in targeting various type of cancer cells. 9-methoxycanthin-6-one is an alkaloid. It may be noted that alkaloids are among the most important active components in natural herbs, and some of these compounds have already been successfully developed into chemotherapeutic drugs, that include paclitaxel, vinblastine, vincristine and camptothecin. Besides these drugs, there are a number of novel alkaloids that have been proven to have anti-proliferative effects on the cancer cells, including berberine, evodiamine, matrine, piperine, sanguinarine, and tetrandrine [30]. 

Colorectal cancer cell lines (HT-29) and ovarian cancer cell lines (A2780, SKOV-3) were used as models to explore the apoptotic mechanism of 9-methoxycanthin-6-one. 9-methoxycanthin-6-one was found to inhibit the cancer cells’ proliferations via apoptosis, when assessed using the Hoechst 33342 assay. Hoechst 33342 is a blue fluorescent dye used to stain DNA as it binds, preferentially to adenine-thymine regions of DNA. Since this fluorescent staining labels DNA, apoptosis from early to late stages occurring within the nucleus can be visualised under a fluorescence microscope [31]. Cells that undergo apoptosis (programmed cell death) have morphology changes, such as chromatin condensation, DNA fragmentation, cell shrinkage, cell membrane blebbing and the formation of apoptotic bodies [4], in which some of these appearances had been visualised when 9-methoxycanthin-6-one was used to treated the HT-29, A2780 and SKOV-3 cancer cells. It was found that the number of apoptotic cells increased when the concentrations of 9-methoxycanthin-6-one increased. It may be noted earlier that many anti-cancer drugs re-induced the apoptotic process in executing cell death. For example, alkaloids, such as paclitaxel, had also been reported to induce apoptotic cell death in A2780 ovarian cancer cell line. Hence, the ability of 9-methoxycanthin-6-one to induce apoptosis may indicate its potential to become an anti-cancer agent by doing further validation studies via in vitro and in vivo tests. 

The recent advances in genomics and proteomics aid the understanding of the true internal mechanisms of carcinogenesis and, also, the biochemical pathways being affected by any potential drug candidate, towards inducing the death of cancer cells. Proteomics tools, such as 2-DE, databases (i.e., Mascot and SwissProt) and softwares (i.e., Image Master Platinium and Ingenuity Pathway Analysis (IPA)) enabled us to analyse the proteins’ expressions and interactions, for at least hundreds of proteins in one go. The mechanisms of the action of anti-cancer activity of 9-methoxycanthine-6-one, when treating ovarian cancer cells, were elucidated by performing the differential analysis between 2-DE gel profiles non-treated and 9-methoxycanthin-6-one-treated A2780 ovarian cancer cells. Based on IPA, 5 out of 15 IDed proteins were found to be involved in the apoptosis signaling pathways as shown in Figure 5. These 5 proteins include 1 enzyme (GAPDH), 1 kinase (PKM), and another 3 other proteins that were not classified elsewhere (ANXA2, HNRNPA1A and LGALS3). The selection was made based on a higher number of selected protein interactions with a known drug target (oncoproteins and tumour suppressor proteins) in the Ingenuity pathway Knowledge Base (IPKB), such as p53 [32], BCL-2, BCL-XL and BAX protein [33,34].

ANXA2 was reportedly playing crucial roles in cancer growth and progression. ANXA2 participation was frequently reported in tumour cell adhesion, proliferation, invasion, metastasis and tumour neovascularisation [35,36]. Based on a previous report, ANXA2 knockdown increased the tumour suppressor protein (p53) activity and downstream gene expression, as well as the p53 translocation from the cytoplasm to the nucleus [36]. p53 protein is the major tumour suppressor and plays an important role in tumour suppression by preventing the division and development of cells with damaged DNA [37,38]. Our findings show that ANXA2 was suppressed in the 9-methoxycanthine-6-one-treated A2780 ovarian cancer cell line. The interference of ANXA2 was reported to increase the expression of p53 [39]. At the same time, LGALS3, an anti-apoptotic protein [40,41] was also found suppressed in this study, and interference of LGALS3 increased the expression of the BAX protein in this pathway. The BAX protein is also known as a apoptosis executioner protein [33,42]. Hypothetically, an increase in the expression of p53 protein leads to the activation of the apoptotic signaling pathway and the death of the apoptotic cell via the activation of the apoptotic regulator, the BAX protein.

BCL-XL and BCL-2 proteins are the family member of the B-cell lymphoma 2 protein (BCL-2), which functioned as anti-apoptotic protein [43,44]. In response to an A2780 ovarian cancer cell line treated with 9-methoxycanthine-6-one, it was noted that 3 proteins, namely GAPDH, HNRNPA1 and PKM, suppressed and interfered with the anti-apoptotic protein, BCL-XL. Suppression of LGALS3 may interfere with the expression of the BCL-2 protein. Interference of BCL-XL and BCL-2 caused an apoptosis-inducing factor (AIF) of the ubiquitination protein in the mitochondria [45] which lead to DNA fragmentation, one of the hallmarks of apoptotic events. The details of the biochemical pathways involved were shown in Figure 5.

To the best of our knowledge, this was the first report of 9-methoxycanthine-6-one from *E. longifolia* affecting the energy pathways such as Glycolysis I and Gluconeogenesis I in ovarian cancer cells. Glycolysis I is a metabolic pathway for energy production in many organisms. A malfunction in energy production can have lethal consequences for the living cells. Cancer cells have a greater need for energy and a ready supply of the building blocks necessary for the synthesis of macromolecules (nucleotides, protein, lipids) in order to duplicate genomes and biomass [46]. Well-known glycolytic enzymes, such as glyceraldehyde-3-phosphate dehydrogenase, also play roles in other cellular processes, such as apoptosis. Furthermore, it has been demonstrated that cell death pathways, particularly proteins that are involved in apoptosis, are closely linked to the cell’s bioenergetic mechanisms [47,48].

Gluconeogenesis I is also a metabolic pathway that results in the generation of glucose from non-carbohydrate carbon substrates, such as pyruvate, lactate, glycerol, glucogenic amino acids, and fatty acids [49]. The purpose of the gluconeogenic pathway is to provide the body with a source of glucose that can be converted to energy for cell survival [50,51]. IPA results showed that the reduction of enzymes involved in Glycolysis I and Gluconeogenesis I could be a contributing factor to ovarian cancer cells failure to proliferate. It may be noted that cancer cells display enhanced rates of glucose uptake and glycolysis. Cancer cells formation starts with cancer cells growing vigorously, and as solid tumours grow, they are unable to obtain oxygen efficiently. In other words, they begin to experience hypoxia [48]. Under these conditions, glycolysis leading to lactic acid fermentation becomes the primary source of energy (ATP) for cancer cells [52]. In this study, proteins that play an important role in pathways of Glycolysis I (GAPDH, PGAM1, PKM and TPI1) and Gluconeogenesis I (GAPDH and PGAM1) were suppressed, except for MDH2 that was expressed in the Gluconeogenesis I pathway. The suppression of these enzymes by 9-methoxycanthin-6-one may affect the energy-producing pathway and lead to the A2780 ovarian cancer cells ceasing to proliferate.

## 4. Materials and Methods

### 4.1. Production of E. longifolia Hairy Roots Culture in the Bioreactor

Approximately 23 g of *E. longifolia* hairy roots from shake flasks were inoculated in the 5 L bioreactor containing 4 litre MS (Murashige & Skoog) basal medium powder with pH 5.8, and maintained in dark condition for 3 months. After 3 months in culture, hairy roots were harvested with fresh weight of about 456 g (20-fold increase) and dried overnight in the oven at 37 °C. Then, the dried roots were ground using pestle and mortar prior to extraction and isolation steps.

### 4.2. Isolation of 9-Methoxycanthin-6-one from E. longifolia Hairy Root Culture

The hairy root powder of *E. longifolia* (96 g) was extracted in chloroform (0.5 L) using soxhlet extraction at 35–45 °C for 16 h. The chloroform solution of extract was filtered and evaporated to yield the chloroform extract (5.3 g, 5.5%). The chloroform extract was fractionated using column chromatography on silica gel 60 (70–230 mesh) using n-hexane, n-hexane-dichloromethane (DCM) (9:1, 7:3, 3:7 and 1:9) as mobile phase to obtain 7 fractions (FR1-FR7). A combined 3 fractions (FR1-FR3) (1.172 g, 1.22%) were then re-chromatographed and purified by column chromatography on silica gel 60 and eluted with n-hexane-DCM (9:1, 7:3, 3:7 and 1:9) and DCM-methanol (1:1) to afford 9-methoxycanthin-6-one (1) (415.8 mg, 0.43%).

### 4.3. Cell Culture and Treatments

The cancer cell lines used for this study were A2780 (ovarian), SKOV-3 (ovarian), MCF-7 (breast), HT-29 (colorectal), HeLa (cervical) and A375 (skin). All cell lines were purchased from American Type Culture Collections (ATCC, Manassas, VA, USA) except for A2780, which was purchased from European Collection of Authenticated Cell Cultures (ECACC, Salisbury, UK). These cells were sub-cultured in Dulbecco’s Modified Eagle’s medium (Sigma-Aldrich, St. Louis, MO, USA) supplemented with 10% fetal bovine serum (Sigma-Aldrich, St. Louis, MO, USA), 1% penicillin-streptomycin (Sigma, USA), 1% amphotericin B (Sigma, USA) and 1% gentamicin (Sigma-Aldrich, St. Louis, MO, USA). The cells were seeded in each well of the 96-well plates and incubated in a humidified incubator at 37 °C and 5% carbon dioxide in air for 24 h. Each cell line was then treated with the 9-methoxycanthin-6-one, isolated from *E. longifolia* at five different concentrations (0.08, 0.4, 2, 10 and 50 µM) in triplicate. Cisplatin and paclitaxel (Sigma-Aldrich, St. Louis, MO, USA), a known chemo-drug, were also treated on these cell lines at five different concentrations (0.08, 0.4, 2, 10, 50 µM for cisplatin and 0.0008, 0.004, 0.02, 0.1, 0.5 µM for paclitaxel) as in the comparative studies. The treated cells were then incubated in the same incubator with the mentioned conditions for 72 h. The experiment was repeated at least three times. 

### 4.4. Cells Viability Assay

Sulphorhodamine B (SRB) assay [53,54] had been performed after the treated cells were incubated for 72 h. Briefly, 50 μL of ice cold tricholoroacetic acid (TCA) was added to each well and allowed to stand for 30 min at room temperature, followed by rinsing each well with tap water. Then, 100 μL of 0.4% SRB was added to each well to stain living cells for 30 min, followed by a rinse with 1% acetic acid. Finally, 100 μL of Tris buffer was added to each well and the optical density (OD) of the treated and non-treated cells were read at 492 nm with a Magellan V.4 microtiter plate reader (Tecan, Salzburg, Austria). The percentage of cell viability was calculated based on (OD_492nm_ of the treated cells/ OD_492nm_ of the non-treated cells) × 100. The IC_50_ values were determined from the dose–response curve of percentage of cell viability versus the concentration of the extracts (µM). Cells’ viability assay for each treatment was performed in triplicate in at least three independent experiments, and the IC_50_ values are given as the mean ± SEM. 

### 4.5. Apoptotic Hoechst 33342 Assay

Approximately 10 × 10^4^ cells/mL were seeded for 500 μL in each four well of three sets of Labtek^TM^ chamber slide (Thermo Fischer Scientific, Waltham, MA, USA), and incubated in 5% carbon dioxide in air for 24 h for cells’ attachment. HT-29, A2780 and SKOV-3 cancer cell lines were selected for determining the apoptotic effect of 9-methoxycanthin-6-one by using Hoechst 33342 assay. The cells were treated with the concentrations of IC_50_/5, IC_50_ and IC_50_ x5 values of 9-methoxycanthin-6-one in HT-29, A2780 and SKOV-3 cancer cell lines. After the treatment of 9-methoxycanthine-6-one, the cells were incubated for 6 h, 24 h and 48 h, respectively. Non-treated cells were also included, which acted as a negative control in this study, in separate chamber per slide. Another two sets of these treated chamber slides were prepared. Each of the chamber slide was incubated in the same condition for 6 h, 24 h and 48 h, respectively. Following this, the cells were washed with cold phosphate buffer solution (PBS) twice and 4% of paraformaldehyde was added in each chamber, that were then further incubated for 30 min. The cells were then washed with PBS, Hoechst 33342 solution (100 μg/mL) was added in each chamber, and they were incubated for 30 min in the dark. Following this, the chambers were discarded and the cells were immediately observed under an inverted fluorescence microscope (BX53, Olympus, Tokyo, Japan) using UV excitation at 200× magnification. Nuclear morphology, including chromatin condensation that is one of the events in apoptotic cells where they will emit fluorescent blue colour when stained with Hoechst 33342 [55]. At least 200 total target cells per area for five areas per chamber were counted, and the numbers of fluorescent and non-fluorescent cells were recorded and analysed using fluorescence microscopy for quantification of the percentage of apoptotic index (% of the number of apoptotic cells/total number of cells). 

### 4.6. Two-Dimensional Gel Electrophoresis (2-DE) 

Ovarian cancer (A2780) cells were seeded at approximately twenty million cells per 75 cm^2^ tissue culture flask. The cells were treated with 9-methoxycanthin-6-one at its IC_50_ value. Non-treated cells were also included in this experiment. Both treated and non-treated cells were incubated for 24 h. Some cells had detached, indicating cell death after the treatment of 9-methoxycanthin-6-one, as compared with the non-treated. Cell pellets were collected and re-suspended in lysis buffer consisting of 6 M urea, 2 M thiourea, 4% CHAPS, 65 mM DTT and protease inhibitor cocktail. Following that, the lysed pellets were then spun at 13,000 rpm for 30 min at 4 °C. The supernatants were then collected and protein contents were estimated using 2D-Quant kit (GE Healthcare, Uppsala, Sweden). The quantification protocol was performed, as described by the manufacturer, using Bovine Serum Albumin (BSA) as protein standard. The absorbance was read at 480 nm. The concentration of protein was estimated from the plotted calibration curve. This quantification was assayed in triplicates. The protein samples were stored at −80 °C until further study. For 2DE profiling, proteins of 200 μg from 9-methoxycanthin-6-one-treated and non-treated ovarian cancer cell lines A2780 were separately mixed with rehydration buffer [6 M urea, 2 M thiourea, 4% CHAPS, 2% Pharmalyte (pH 3–10)]. Then the Immobiline IPG DryStrips (11 cm, pH 3–10) (GE Healthcare, Uppsala, Sweden) were re-swelled in the mixtures and rehydrated overnight. The swollen IPG Strips were then isoelectrically focused at 300 V for 1 h, 12,000 V/hr on an IPGphor Electrophoresis system (Bio-Rad, Hercules, CA, USA). After equilibration for 2 × 15 min in equilibration buffer, IPG strips were applied for 12% Sodium Dodecyl Sulphate-Poly Acrylamide Gel Electrophoresis (SDS-PAGE) using a SE 600 Ruby (GE Healthcare, Uppsala, Sweden). 

### 4.7. Image and Data Analysis 

The gels were stained using Coomassie Brilliant Blue G-250 (GE Healthcare, Uppsala, Sweden). The protein spots were detected, quantified, and matched using ImageMaster 2D Platinum 7.0 analysis software (GE Healthcare, Uppsala, Sweden). Differences in the expression levels between paired samples were analysed, and protein spots showing at least 1.5-fold changes in intensity and anova *p* < 0.05 were picked for trypsin digestion and MALDI-TOF MS analysis.

### 4.8. In-Gel Tryptic Digestion 

Protein samples were trypsin digested and peptides were extracted, according to [56]. Briefly, gel samples were de-stained with ammonium bicarbonate/acetonitrile, then underwent a 16 h tryptic digest at 37 °C in 25 mM ammonium bicarbonate (10 μL). Peptides were extracted and concentrated by C18 ziptip, spotted onto MALDI AnchorChip plate with 1 μL CHCA matrix. Matrix Assisted Laser Desorption Ionisation (MALDI) mass spectroscopy was performed using the 4800 plus MALDI TOF/TOF Analyser. An Nd:YAG laser (355 nm) was used to irradiate the sample. Spectra were acquired in reflectron MS scan mode in the mass range of 700 to 4000 Da. The instrument was then switched to MS/MS mode, where the eight or twelve strongest peptides from the MS scan were isolated and fragmented by collision-induced dissociation, then re-accelerated to measure their masses and intensities. A near point calibration was applied and which gives a typical mass accuracy of 50 ppm or better. Spectra were analysed to identify proteins of interest using Mascot (matrix Science Ltd., London, UK) database search and UniProt database.

### 4.9. Mass Spectrometry and Database Search 

The proteomics dataset, which included UniProt identifiers and fold changes of differentially expressed proteins, was submitted to Ingenuity Pathways Analysis (IPA) for core analysis. The core analysis was performed using settings in Ingenuity Knowledge Base for direct and indirect relationships between molecules, based on experimental data and IPA data sources. IPA was then used to build protein interactions, pathways, and functional network relationships (Qiagen, Hilden, Germany). The modulated proteins were grouped by known relationships into the “Functional Analysis” and “Canonical Pathway Analysis” of a network, with significance calculated using Fisher’s exact test (*p*-value ≤ 0.05) to determine the probability of each biological function and/or disease. For the IPA network analysis, hypothetical protein interaction clusters between the molecules were analysed. IPA computes a *p*-score to rank networks according to the differentially expressed proteins. The score was calculated as *p*-score = −log 10 (*p*-value), which indicates the probability of matching the input proteins in a protein–protein interaction from the Ingenuity Knowledgebase by random chance. A score of 3 or higher indicates at least a 99.9% confidence level for excluding random chance. Based on this, identified proteins were connected with hub proteins, forming a functional protein cluster.

## 5. Conclusions

In conclusion, the results obtained had confirmed the in vitro anti-cancer effect of 9-methoxycanthin-6-one in the listed cancer cell lines. 9-methoxycanthin-6-one showed potential in inducing apoptosis in a dose-dependent manner. Proteins that were identified in this apoptotic event when the A2780 ovarian cancer cells were treated with 9-methoxycanthin-6-one were Pyruvate kinase (PKM), annexin A2 (ANXA2), galectin 3 (LGAL3), heterogeneous nuclear ribonucleoprotein A1 (HNRNP1A1), peroxiredoxin 3 (PRDX3) and glyceraldehyde-3-phosphate dehydrogenase (GAPDH). Besides the apoptosis pathway, 9-methoxycanthine-6-one may also effect the Glycolysis I and Gluconeogenesis I pathways in ovarian cancer cells. Validation studies will be conducted in the near future on these mentioned proteins using the western blotting technique.

## 6. Patents

Yunos, N.M. et al. Combination of therapeutic agents for treating ovarian cancer. Malaysia Patent Filed PI2016701034. Intellectual Property Corporation of Malaysia (MYiPO). 23 March 2016. 

## Figures and Tables

**Table 1:** ^1^H and ^13^C-NMR spectral data of 9-methoxychanthin-6-one (1) (400, 100 MHz in CDCl_3_).

No	^1^H δ ppm	^13^C δ ppm
1	8.23 (1H, d)	115.660
2	8.78 (1H, d)	143.810
3	-	-
4	7.98 (1H, d)	138.053
5	6.99 (1H, d)	129.685
6	-	159.376
7	-	-
8	7.11 (1H, d)	101.448
9	-	163.411
10	7.91 (1H, d)	114.756
11	8.12 (1H, d)	123.949
12	-	116.775
13	-	142.084
14	-	129.691
15	-	131.150
16	-	132.362
OCH_3_	4.04 (3H, s)	56.200

**Table 2:** The IC_50_ values (μM) ± SEM (*n* = 9) of 9-methoxycanthin-6-one (EL50), cisplatin (Cis) and paclitaxel (Tax), tested in a panel of cancer cell lines (ovarian cancer (A2780, SKOV-3), breast cancer (MCF-7), colorectal cancer (HT29), cervical cancer (HeLa) and skin cancer (A375)) and a normal cardiomyocyte cell line (H9C2).

Compound	A2780	SKOV-3	MCF-7	HT-29	A375	HeLa	H9c2
EL50	4.04 ± 0.36	5.80 ± 0.40	15.09 ± 0.99	3.79 ± 0.069	5.71 ± 0.20	4.30 ± 0.27	37.34 ± 0.91
Cis	1.77 ± 0.018	2.27 ± 0.10	3.58 ± 0.14	1.38 ± 0.037	2.42 ± 0.19	1.54 ± 0.12	25.27 ± 0.77
Tax	0.018 ± 0.00011	0.38 ± 0.012	>0.5	0.35 ± 0.0040	0.37 ± 0.0055	0.31 ± 0.0078	>0.5

Note: SEM (Standard Error of the Mean) < 5%.

**Table 3:** The percentage of apoptotic indices of 9-methoxycanthin-6-one when treated in HT-29, A2780 and SKOV-3 cancer cell lines at different concentrations and incubation times.

Cell Line	9-Methoxycanthin-6-one (µM)	Apoptotic Index (%)		
		6 h	24 h	48 h
HT-29	Non-treated	1.76 ± 0.36	1.68 ± 0.38	1.81 ± 0.32
	IC_50_/5	3.36 ± 0.76 ^b^	3.88 ± 1.04 ^b^	4.14 ± 0.25 ^c^
	IC_50_	4.23 ± 0.35 ^c^	4.56 ± 0.31 ^c^	5.31 ± 0.76 ^d^
	IC_50_x5	100.00 ± 0.00 ^d^	100.00 ± 0.00 ^d^	100.00 ± 0.00 ^d^
A2780	Non-treated	2.26 ± 0.39	2.62 ± 0.61	2.71 ± 0.11
	IC_50_/5	3.85 ± 0.08 ^c^	4.13 ± 0.68	4.27 ± 0.58 ^a^
	IC_50_	4.49 ± 0.35 ^d^	5.34 ± 1.45 ^a^	7.04 ± 1.14 ^c,w^
	IC_50_x5	100.00 ± 0.00 ^d^	100.00 ± 0.00 ^d^	No cells were detected
SKOV-3	Non-treated	2.05 ± 0.56	2.07 ± 0.54	1.65 ± 0.35
	IC_50_/5	2.10 ± 0.19	2.33 ± 0.64	2.42 ± 0.16 ^a^
	IC_50_	3.38 ± 0.69 ^a^	4.86 ± 0.09 ^d,w^	11.20 ± 0.39 ^d,z^
	IC_50_x5	6.30 ± 0.22 ^d^	100.00 ± 0.00 ^d,z^	100.00 ± 0.00 ^d,z^

Statistical analysis was performed using one-way analysis of variance (ANOVA) followed by Dunnett’s comparison test using Graphpad Prism version 7. ^a,b,c,d^ letters represent significant differences within the groups when compared to non-treated (dose-dependent experiment) while ^w,z^ letters represent significant differences between the groups when compared to 6 h (time-dependent experiment). ^a^ and ^w^ letters refer to *p* < 0.05; ^b^ letter refers to *p* < 0.01; ^c^ letter refers to *p* < 0.001; ^d^ and ^z^ letters refer to *p* < 0.0001.

**Table 4:** Identification of differentially expressed proteins induced by 9-methoxycanthine-6-one when treated in A2780 cancer cell line. The information listed in this Table was then subjected for the Ingenuity Pathway Analysis (IPA) for protein–protein interactions studies.

No	Protein Name ^a^	Gene Name	Accession No ^b^	pIc	mW (Da) ^c^	Sequence Coverage (%)	Mascot Score	Protein Abundance
1	ALDH1A1	Aldehyde dehydrogenase 1 family member A1	P00352	6.3	54,827	30%	233	−1.52
2	PKM	Pyruvate kinase isozymes M1/M2	P14618	7.96	57,900	54%	413	−2.11
3	EEF1A1	Eukaryotic translation elongation factor 1 alpha 1	P68104	9.10	50,109	33%	153	−4.29
4	LGALS3	Galectin-3	P17931	8.57	26,136	27%	235	−6.23
5	CAPG	Capping actin protein	P40121	5.82	38,474	29%	167	−2.35
6	ANXA2	Annexin A2	P07355	7.57	38,580	43%	431	−5.72
7	NVL	Nuclear valosin-containing protein-like	O15381	6.11	94,991	28%	64	+8.85
8	PNP	Purine nucleoside phosphorylase	P00491	6.45	32,097	42%	93	−3.78
9	PGAM1	Phosphoglycerate mutase 1	P18669	6.67	28,786	57%	583	−2.94
10	TPI1	Triosephosphate isomerase 1	P60174	5.65	30,772	59%	556	−2.87
11	PRDX3	Thioredoxin-dependent peroxide reductase	P30048	7.67	27,675	25%	128	−2.80
12	HNRNPA1	Heterogeneous nuclear ribonucleoprotein A1	P09651	9.17	38,723	45%	195	−14.65
13	GAPDH	Glyceraldehyde-3-phosphate dehydrogenase	P04406	8.57	36,030	22%	239	−4.29
14	MDH2	Malate dehydrogenase	P40926	8.92	35,481	39%	278	+4.00
15	ACAA2	Acetyl-CoA acyltransferase 2	P42765	8.32	41,898	28%	128	+4.8

^a^ Measured peptide and fragment ion masses were used to search against the Swiss-Prot/Mascot databases for protein identifications using the Mascot software. Ion scores (based on mass/mass spectrums) were from MALDI-TOF/TOF identification. A Mascot score of >56 was considered as identity (*p* < 0.05). ^b^ Accession numbers were derived from the Mascot database. ^c^ Theoretical molecular weight (MW) or isoelectric point (pI) from the Mascot database. Proteins with negative (−) value indicate suppressed proteins, while proteins with positive (+) value indicate expressed protein.

**Table 5:** Top canonical pathways involved in the mechanism of action of 9-methoxycanthine-6-one when treated in A2780 ovarian cancer cell line.

No	Canonical Pathway	*p*-Value	Proteins Involved
1	Glycolysis I	9.38 × 10^−9^	GAPDH, PGAM1, PKM, TPI1
2	Gluconeogenesis I	2.62 × 10^−6^	GAPDH, PGAM1, MDH2,
3	NADH Repair	4.69 × 10^−3^	GAPDH
4	Xanthine and Xanthosine Salvage	5.27 × 10^−3^	PNP
5	Guanine and Guanosine Salvage I	5.86 × 10^−3^	PNP

## Data Availability

All relevant data are already included in the text.

## Sample Availability

Samples of the compound 9-methoxycanthin-6-one and the extract are available from the authors.
